# Enhanced Recovery After Surgery (ERAS): Transforming Orthopedic Trauma Management

**DOI:** 10.7759/cureus.99453

**Published:** 2025-12-17

**Authors:** Ahmed Mohamed, Daniel Francis, Usman Fuad, Sagaurav Shrestha, Tamaraebi Itoko, Ioannis P Pengas

**Affiliations:** 1 Trauma and Orthopaedics, Royal Cornwall Hospital, Truro, GBR

**Keywords:** enhanced recovery after surgery, eras, hip fracture, multidisciplinary care, orthopedic trauma, perioperative care

## Abstract

Enhanced Recovery After Surgery (ERAS) protocols represent a paradigm shift in perioperative care, emphasizing evidence-based multidisciplinary approaches to optimize surgical outcomes. This narrative review examines the current evidence for ERAS implementation in orthopedic trauma care by synthesizing literature from major medical databases. The ERAS protocols in patients with hip fractures demonstrate robust evidence for improved outcomes, including reduced hospital length of stay, decreased complication rates, lower delirium and infection rates, and improved pain control with reduced opioid consumption. Emerging evidence for ankle and other extremity fractures shows similar benefits, including a faster return to function and improved patient satisfaction. The key ERAS components include preoperative optimization, intraoperative stress reduction, and aggressive postoperative mobilization. Despite compelling evidence, implementation remains challenging because of resource limitations, knowledge gaps, organizational barriers, and the unpredictable nature of trauma care. Successful ERAS programs require dedicated multidisciplinary teams, institutional commitment, clear protocols, ongoing education, and robust measurement systems. As the evidence base expands and implementation science advances, ERAS has the potential to become the standard of care for trauma patients worldwide, with future directions including technology integration, precision medicine approaches, and adaptation to resource-limited settings to further enhance the reach and effectiveness of these protocols.

## Introduction and background

Surgery has changed dramatically over the past few decades. Physicians have progressively transitioned from traditional models of care to patient-centered approaches guided by evidence-based medicine. One of the most important changes is the Enhanced Recovery After Surgery (ERAS) program, a comprehensive care system that has improved patient recovery from surgery. A Danish surgeon named Henrik Kehlet created ERAS in 1997 to be used in bowel surgery. Since then, ERAS has been implemented in many surgical fields. The program helps patients recover faster, experience fewer complications, and feel more satisfied with their care [1].

ERAS works effectively in elective operations, such as joint replacements; however, using ERAS for trauma patients poses more challenges. Trauma patients arrive at hospitals without warning, often with significant injuries and preexisting health problems. Although there is less time to prepare trauma patients for surgery than for elective surgery, trauma patients also benefit greatly from organized, evidence-based care [2].

This review examines the use of ERAS in orthopedic trauma. We examine how these programs function in emergency settings. We reviewed the results that doctors observed. We discuss the remaining challenges. This review covers the main components of ERAS programs, the research supporting them, and the real-world difficulties in implementing these programs in busy trauma centers.

## Review

Methodology

This narrative review synthesizes current evidence on ERAS protocols in orthopedic trauma to provide clinicians and researchers with a comprehensive understanding of implementation strategies, outcomes, and future directions. We chose a narrative review approach rather than a systematic review due to the substantial heterogeneity in study designs, ERAS protocol components, patient populations, and outcome measures across the orthopedic trauma literature, which precludes meaningful quantitative synthesis. Additionally, our goal was to provide a broad overview encompassing clinical evidence, implementation science, and practical guidance rather than answering a narrow, focused clinical question.

We conducted a comprehensive literature search of PubMed, EMBASE, and Cochrane Library databases for English-language publications from January 2015 through October 2025. Search terms included “Enhanced Recovery After Surgery,” “ERAS,” “fast-track surgery,” “orthopedic trauma,” “hip fracture,” “extremity trauma,” “perioperative care,” and “multimodal analgesia.”

We prioritized high-quality evidence, including systematic reviews and meta-analyses, randomized controlled trials, large cohort studies (n > 100), clinical practice guidelines, and implementation science studies examining barriers, facilitators, and audit outcomes. Studies were included if they examined ERAS protocols in orthopedic trauma populations and reported clinical outcomes (length of stay, complications, pain, function, mortality, readmissions) or implementation outcomes (adherence, barriers, cost-effectiveness). Studies focusing exclusively on elective orthopedic surgery without trauma applications were excluded.

Understanding ERAS: core principles and components

Enhanced recovery pathways transform our approach to surgical care by treating recovery as an active, optimizable process rather than passive healing. ERAS targets two main impediments to recovery: surgical stress and organ dysfunction. By systematically addressing these through evidence-based interventions across the perioperative continuum, patients achieve faster recovery, fewer complications, and earlier return to function [3,4].

Enhanced recovery programs span three phases: preoperative, intraoperative, and postoperative. Each contains multiple coordinated interventions delivered by multidisciplinary teams. The core components are summarized in Table 1. The strength of ERAS lies not in individual interventions but in their synergistic integration as a cohesive care system [4,5].

**Table 1:** Core components of Enhanced Recovery After Surgery (ERAS) protocols in orthopedic trauma. This table is derived from references [6-26]. NSAIDs: nonsteroidal anti-inflammatory drugs; PONV: postoperative nausea and vomiting

Phase	Key components
Preoperative	• Patient and family education • Medical optimization and multidisciplinary team activation • Abbreviated fasting protocols (clear fluids up to 2 hours pre-surgery) • Multimodal analgesia (regional nerve blocks, NSAIDs, minimal opioids)
Intraoperative	• Regional anesthesia techniques (spinal preferred for hip fractures) • Peripheral nerve blocks based on fracture location • Active normothermia maintenance • Goal-directed fluid management • Blood conservation strategies
Postoperative	• Early mobilization (chair day 0, walking within 24 hours) • Multimodal pain management with minimal opioids • Early nutrition • PONV prevention • Delirium prevention (especially for elderly patients) • Early catheter and drain removal • Thromboprophylaxis

To truly understand how ERAS works in trauma, we need to examine individual components more closely. While specific protocols vary between institutions and injury types, most ERAS pathways share common elements proven to improve outcomes.

Preoperative Phase

The preoperative phase differs markedly between trauma and elective patients. Elective patients might have weeks to prepare. Trauma patients often have only hours. Despite these constraints, several ERAS principles can still be applied effectively.

Patient and family education begins immediately upon arrival. Even in emergency settings, taking time to explain what will happen, setting expectations, and addressing concerns reduces anxiety and improves cooperation with the care plan. This psychological preparation, often overlooked in the rush of trauma care, can significantly impact recovery [6].

Medical optimization proceeds systematically. The trauma team assesses and stabilizes life-threatening conditions, optimizes fluid status, corrects coagulopathy, and manages pain effectively. For hip fracture patients specifically, this phase involves immediate multidisciplinary team (MDT) activation, including orthopedic surgeons, geriatricians, anesthetists, nurses, physical therapists, and occupational therapists. Each plays a crucial role in optimizing the patient for surgery, as delays worsen outcomes, particularly in elderly populations [7].

Fasting guidelines have significantly evolved under ERAS. Traditional rules forbidding eating or drinking after midnight are no longer routinely applied. Patients can drink clear fluids up to two hours before surgery. This helps prevent dehydration and may reduce insulin resistance, which is a key component of surgical stress. Although trauma patients do not always have this opportunity, applying these principles when safe to do so still yields benefits [8].

Multimodal pain management begins before surgery. Rather than waiting until the pain becomes severe, enhanced recovery protocols advocate proactive pain management using multiple medications that work through different mechanisms. This includes anti-inflammatory drugs when not contraindicated, regional nerve blocks, and minimal use of opioids. Fascia iliaca nerve blocks are commonly employed in patients with hip fractures, providing excellent pain relief while avoiding the cognitive side effects of opioids that can precipitate delirium in elderly patients. The goal is preventing pain rather than chasing it, thereby reducing overall opioid requirements and associated side effects [9,10].

Intraoperative Phase

During surgery, ERAS principles focus on minimizing the physiological impact of the procedure. Anesthetic management is carefully tailored, with a preference for regional techniques when appropriate. Spinal anesthesia is often preferred for hip fractures when feasible, as it is associated with reduced delirium compared to general anesthesia. However, this decision must be individualized based on patient factors, injury severity, and anticipated surgical duration [9].

Local anesthetic infiltration and peripheral nerve blocks are the cornerstones of intraoperative pain management. These techniques provide effective analgesia, allowing patients to awaken comfortably, mobilize early, and reduce their opioid requirements. The commonly used blocks for upper limb fractures include interscalene, supraclavicular, infraclavicular, and axillary brachial plexus blocks. For lower limb fractures, options include femoral, fascia iliaca, adductor canal, sciatic, and popliteal nerve blocks, selected according to the fracture location and surgical approach. Local wound infiltration or digital blocks are also important when regional anesthesia cannot be performed [11-13].

Maintaining a normal body temperature is crucial. Even mild hypothermia can increase blood loss, impair coagulation, increase the risk of infection, and delay drug metabolism. Enhanced recovery protocols emphasize active warming measures, including warm operating rooms, forced-air warming blankets, warmed intravenous fluids, and heated humidified gases for intubated patients. This is particularly important in hip fracture surgery, where elderly patients are at an increased risk of hypothermia [14].

Fluid management aims to avoid both inadequate and excessive fluid administration. Excessive fluid can cause tissue edema, delayed wound healing, and prolonged gut dysfunction in patients with abdominal trauma. Conversely, inadequate fluid intake impairs tissue perfusion and organ function. Modern ERAS protocols use various monitoring techniques to guide fluid administration and ensure that patients receive optimal amounts. These include arterial waveform analysis, which measures stroke volume and pulse pressure variation to assess fluid responsiveness; esophageal Doppler monitoring, which provides real-time data on blood flow and cardiac output; and noninvasive cardiac output monitors that estimate hemodynamic status using finger-cuff or bioreactance technology. Lactate levels and base deficit trends are also used as indicators of tissue perfusion, whereas point-of-care ultrasound helps assess intravascular volume and cardiac function at the bedside [15,16].

Blood conservation strategies are important in trauma, where significant hemorrhage is common. These include meticulous surgical techniques, the use of tranexamic acid to reduce bleeding, appropriate transfusion thresholds, and cell salvage techniques. For hip fractures, surgical approaches that minimize blood loss and reduce operative time are prioritized, as prolonged procedures increase complications in older adult patients. The goal is to minimize both bleeding and unnecessary blood transfusions [17].

Postoperative Phase

The postoperative phase is the area in which ERAS protocols have the greatest impact. Early mobilization is the most impactful and challenging component of rehabilitation. Getting patients out of bed and moving within hours of surgery contradicts traditional bed rest approaches; however, evidence strongly supports this practice. Prolonged immobility triggers a cascade of negative effects: muscle wasting begins within hours, bone density reduces, thromboembolic risk increases, pressure ulcer and hospital-acquired infection risks rise, lung function deteriorates, and delirium becomes more likely. For elderly patients with hip fractures, each day of immobility increases the risk of never regaining pre-fracture functional status. Enhanced recovery protocols aim to have patients sitting in a chair on the day of surgery and taking their first steps within 24 hours, supported by physical therapy teams who understand that carefully supervised early mobilization is safe and beneficial [18,19].

Pain management continues with the multimodal approach established preoperatively. The key is to stay ahead of pain using scheduled doses of non-opioid analgesics rather than waiting for patient requests. This typically includes a combination of regional techniques (such as continuous nerve blocks via catheters), non-opioid medications such as acetaminophen and anti-inflammatory drugs, and judicious use of opioids only when necessary for breakthrough pain. Regional anesthesia blocks may be maintained with catheters that provide continuous infusion. Opioids are used sparingly as they carry significant risks in trauma patients, particularly in elderly individuals, including constipation, urinary retention, delirium, and respiratory depression [20].

Nutritional support is initiated early. Traditional practices of withholding food from patients until bowel sounds return have been abandoned in favor of early oral intake. For most trauma patients, oral intake can be safely resumed once they are fully awake and alert. Early nutritional support is a critical component of recovery, as adequate protein intake promotes wound healing and preserves lean body mass, and carbohydrates help combat the catabolic stress response associated with surgery [21,22].

The prevention of postoperative nausea and vomiting has received systematic attention in enhanced recovery protocols. Nausea and vomiting are unpleasant, prevent early mobilization and oral intake, increase pain, and can lead to aspiration. Multimodal prevention using combinations of antiemetic medications from different drug classes has proven highly effective for nausea prevention [23].

Delirium prevention is crucial in older adult trauma patients, particularly those with hip fractures. ERAS protocols incorporate multiple strategies, including minimizing opioids and other confusion-inducing medications, ensuring adequate pain control as pain can trigger delirium, optimizing sleep with appropriate lighting and noise control, encouraging family presence, ensuring patients have glasses and hearing aids, and regular screening for early delirium signs. Environmental optimization, sleep hygiene, and minimizing unnecessary interventions, such as urinary catheters, all contribute to delirium prevention. Teams often use validated screening tools to detect delirium early, when interventions are most effective [24].

Catheter and drain management follows the “as little as necessary, as briefly as possible” philosophy. Urinary catheters are removed as soon as patients can safely mobilize to the bathroom, as prolonged catheterization increases the infection risk and impedes mobilization. Similarly, surgical drains are avoided when possible or removed early, as evidence suggests that they do not prevent hematoma formation but create patient discomfort and immobility [25].

Thromboprophylaxis requires careful balancing in trauma patients. Mechanical prevention with compression devices begins immediately. Pharmacologic prevention with anticoagulants is initiated once the bleeding risk decreases and when full mobility is delayed. This is particularly critical in patients with hip fractures, where thromboembolic events can be fatal [26].

ERAS applications in trauma and their outcomes

Hip Fractures: The Strongest Evidence Base

Hip fractures in elderly patients represent the most common and well-studied application of ERAS in trauma care. With 7.3 to 21.3 million cases projected worldwide by 2050, hip fractures pose substantial morbidity and mortality risks [27]. Studies document 6-9% mortality within 30 days and 21-30% within one year, alongside high rates of delirium, infections, thromboembolic events, and functional decline [28,29]. This devastating clinical picture makes hip fractures an ideal target for systematic perioperative optimization. Professional societies, including the British Orthopaedic Association and the American Academy of Orthopaedic Surgeons, have recognized this opportunity, establishing standardized care frameworks that align with ERAS principles and emphasize multidisciplinary pathways incorporating enhanced recovery elements [30,31].

The key evidence for hip fracture ERAS comes from Liu et al.’s (2021) meta-analysis of seven cohort studies involving 9,869 patients. This comprehensive analysis demonstrated that ERAS programs significantly reduced hospital length of stay by a mean of 2.64 days (95% confidence interval (CI) = -3.63 to -1.65). Beyond shorter hospital stays, ERAS implementation was associated with lower overall complication rates (odds ratio (OR) = 0.68, 95% CI = 0.57-0.80), significant reductions in delirium (OR = 0.46), and decreased urinary tract infections (OR = 0.39). Importantly, these benefits did not compromise patient safety; ERAS had no significant effect on 30-day readmission rates (OR = 1.09, 95% CI = 0.97-1.24) or mortality at 30 days (OR = 0.84, 95% CI = 0.67-1.06) and one year (OR = 0.99, 95% CI = 0.87-1.14). These results indicate that accelerated discharge did not simply shift complications from inpatient to outpatient settings [32].

More recent studies continue to refine our understanding of ERAS implementation in hip fractures. Chen et al. (2025) retrospectively analyzed 120 elderly hip fracture patients, comparing 62 patients managed with an ERAS-based MDT model to 58 receiving conventional care. The ERAS-MDT group achieved significantly earlier mobilization (16.55 ± 1.17 hours) and longer daily walking duration. Postoperative pain was lower, with greater reductions in Visual Analog Scale (VAS) scores by day seven. Functional outcomes favored the ERAS group, with higher Harris Hip Function scores at all follow-up points and more pronounced improvements in daily living abilities. Psychological well-being also improved, as indicated by lower Symptom Checklist-90 symptom-distress scores at discharge. Importantly, in-hospital complication rates were significantly lower in the ERAS-MDT group, demonstrating the safety and efficacy of the ERAS-MDT approach [33].

Large-scale registry data provide real-world validation of these findings. The UK National Hip Fracture Database, analyzing over 50,000 patients across multiple institutions, demonstrated that hospitals with comprehensive ERAS-aligned pathways achieved significantly better outcomes, including reduced 30-day mortality, shorter length of stay, and improved early mobilization rates [34]. This registry evidence is particularly valuable as it reflects implementation across diverse healthcare settings rather than just research-focused academic centers.

Collectively, the hip fracture literature demonstrates that ERAS is feasible, safe, and effective in elderly trauma patients despite the challenges of emergency presentation, complex comorbidities, and limited time for preoperative optimization. The consistency of benefits across meta-analyses, individual trials, and large registries provides robust evidence supporting ERAS as the standard of care for hip fracture management.

Ankle and Extremity Fractures: Emerging Evidence

Beyond hip fractures, ERAS application to extremity trauma shows promise, though the evidence base remains more limited. Ankle fractures represent a particularly relevant application given their frequency (they are among the most common lower extremity injuries) and their significant impact on functional recovery, work ability, and activities of daily living. Unlike hip fractures, which predominantly affect elderly patients, ankle fractures occur across all age groups, making return to work and recreational activities key outcome priorities [35].

Yao et al. (2023) conducted a retrospective comparison of 160 patients undergoing operative fixation for ankle fractures, comparing ERAS versus conventional pathways. The ERAS pathway demonstrated significant advantages in healthcare efficiency, with shorter hospital length of stay (p < 0.01) and substantially lower hospital costs, showing a mean difference of approximately ¥6,501.81 (95% CI = -10,955.21 to -2,048.42, p < 0.01) in favor of ERAS. Beyond economic benefits, functional recovery outcomes favored the ERAS group. At three months postoperatively, ERAS patients showed improved functional recovery (p < 0.001), with this benefit persisting at six months (p < 0.001). Interestingly, by 12 and 24 months, functional differences between groups were no longer statistically significant, suggesting that ERAS primarily accelerates early recovery rather than changing ultimate outcomes, a clinically meaningful finding as faster recovery allows earlier return to work and daily activities [36].

Yang et al. (2025) examined 376 patients undergoing ankle fracture surgery, comparing ERAS and conventional care. ERAS patients experienced significantly lower postoperative pain at multiple time points (24 hours, 48 hours, and 7 days), reflecting the effectiveness of multimodal analgesia. Early functional recovery was markedly accelerated: ERAS patients ambulated significantly earlier (20.4 ± 5.7 hours versus 42.8 ± 6.9 hours) and achieved independent walking sooner (3.6 ± 1.1 days versus 5.9 ± 1.4 days). Quality of life outcomes were superior in the ERAS group, with higher Short-Form 36 and EuroQol-5 Dimensions/EuroQol VAS scores. A greater proportion of ERAS patients returned to work or daily routines by follow-up (81.6% versus 68.7%) and attained independence in activities of daily living (89.5% versus 75.8%) [37].

These ankle fracture findings are encouraging because they suggest that ERAS principles are broadly applicable across various extremity trauma types. The consistent benefits in pain control, hospital efficiency, early functional recovery, and patient satisfaction mirror those seen in hip fractures, despite the very different patient demographics and clinical priorities. However, important caveats remain. Both studies were single-center retrospective analyses from specialized institutions, limiting generalizability. Protocol components varied between studies, making it difficult to identify which specific ERAS elements are most critical for ankle fractures. Long-term outcomes beyond one year remain understudied.

Despite differences in patient populations and injury patterns, several consistent themes emerge across the orthopedic trauma ERAS literature. Hospital length of stay reductions appear universal, ranging from 0.5 to 3 days depending on injury type and baseline institutional practices. Pain control improves consistently with multimodal analgesia approaches, reducing opioid requirements, a particularly important benefit given ongoing concerns about opioid-related complications and the opioid crisis more broadly. Early functional recovery accelerates across all studied injury types, whether measured by time to mobilization, return to independent activities of daily living, or return to work. Complication rates decrease without increasing readmissions or mortality, demonstrating that ERAS enhances rather than compromises safety. Patient satisfaction and quality of life measures consistently favor ERAS, reflecting alignment between enhanced recovery principles and patient priorities [37,38].

Cost-effectiveness data, though limited, suggest that ERAS implementation can yield substantial net financial savings despite requiring upfront investment in infrastructure, training, and resources. Stone et al. (2015) modeled the costs of implementing a US-based ERAS program and found that the initial implementation cost of $552,783 was offset by first-year savings of nearly $948,500, resulting in net savings of $395,717. Reductions in hospital length of stay (0.7-2.7 days) were the primary driver of these savings. Sensitivity analyses across various caseload and direct cost scenarios confirmed that ERAS programs can be economically advantageous for hospitals, supporting institutional investment in these protocols [39].

Critical questions persist: Which specific ERAS components are most important for different extremity injury types? Can these protocols be adapted for patients with multiple traumatic injuries? How can ERAS be implemented effectively in community hospitals with limited resources? What is the optimal approach for polytrauma patients requiring staged procedures? These questions represent both challenges and opportunities for future research and quality improvement [40]. The current evidence provides proof of concept that ERAS benefits extend beyond hip fractures, but substantially more research is needed before evidence-based, injury-specific guidelines can be developed for the full spectrum of extremity trauma.

Implementation challenges: the reality on the ground

Despite compelling evidence, the implementation of ERAS in trauma faces significant challenges. Understanding these barriers is essential for anyone hoping to establish or improve an enhanced recovery program.

Perhaps the most fundamental challenge is the emergency nature of trauma care. Trauma patients arrive unexpectedly, often outside regular working hours. Creating infrastructure for 24-hour ERAS delivery with immediate access to regional anesthesia, physical therapy, appropriate pain medication protocols, and coordinated multidisciplinary care requires a substantial organizational commitment [41].

Knowledge gaps also represent a significant barrier. A survey of healthcare providers in France found ERAS practice remained limited, with only about one-fifth of respondents using it. The study identified a lack of awareness of the existence of ERAS guidelines for specific surgeries and an insufficient understanding of enhanced recovery principles as major barriers. Even in centers of excellence with high buy-in for ERAS, compliance remains moderate. This suggests that awareness and education require substantial improvement [42].

Cultural and organizational barriers often impede the adoption of new practices within healthcare systems, which tend to be inherently conservative. The principle of “first, do no harm” can inadvertently reinforce the reliance on traditional methods, even when emerging evidence supports superior alternatives. Encouraging surgeons, anesthetists, nurses, therapists, and other healthcare professionals to replace familiar routines with evidence-based protocols requires sustained leadership, targeted education, and a clear demonstration of improved outcomes. Hierarchical medical structures can make change difficult when not all team members are equally committed [43].

Multidisciplinary coordination represents both the greatest strength and most challenging aspect of ERAS. Enhanced recovery is not something that can be implemented by a single person or discipline. This requires orchestrated efforts across multiple teams, each with its own priorities, workflows, and schedules. Creating communication channels, protocols, and a culture to support this coordination requires time and persistent effort. Regular multidisciplinary meetings, shared electronic health records, standardized order sets, and clear role definitions all help, but building this infrastructure is challenging [44].

Standardization versus individualization creates an ongoing tension. ERAS protocols work best when standardized, with everyone following the same evidence-based pathway. However, trauma patients are heterogeneous with varying injuries, comorbidities, and social situations. Finding the balance between protocol-driven care and individualized medicine requires clinical judgment and flexibility. Some patients cannot follow standard pathways due to specific contraindications or complications. Protocols must accommodate these variations without abandoning the core principles [45,46].

Measurement and feedback systems are essential for sustained ERAS success, but require infrastructure that many institutions lack. Knowing whether enhanced recovery is working requires tracking and auditing relevant outcomes, including length of stay, complications, readmissions, patient satisfaction, and protocol compliance. Analyzing these data, identifying improvement areas, and adjusting protocols accordingly require dedicated teams and data systems. Without this feedback loop, ERAS programs can drift from evidence-based practice over time [47].

The lack of human and financial resources represents a practical challenge in community hospitals and resource-limited settings. While ERAS reduces costs through shorter hospital stays and fewer complications, its implementation requires an upfront investment. Successful implementation requires access to specific resources, including regional anesthesia expertise available around the clock, adequate physical therapy staffing to support early mobilization, availability of specific medications and equipment, and sufficient nursing staff to manage the increased demands of enhanced recovery protocols. Many institutions lack these resources or struggle to allocate them appropriately [46,48].

Future directions and emerging opportunities

Several exciting developments promise to advance ERAS in trauma. Enhanced recovery is not a static concept but an evolving field that continuously incorporates new evidence and innovation.

The integration of technology holds substantial promise for advancing the implementation of ERAS. Digital health innovations, such as smartphone applications for patient education and monitoring, wearable devices that track physical activity and vital signs, and telehealth platforms enabling remote assessment and follow-up, have the potential to extend ERAS principles beyond the hospital setting. For example, a hip fracture patient could have their postoperative mobility remotely monitored, triggering automatic alerts for concerning trends, while an ankle fracture patient might engage in daily digital check-ins with the care team to facilitate early identification of complications. Such technologies could enhance accessibility, improve outcomes, and optimize resource utilization in enhanced recovery programs [49,50].

Precision medicine approaches may allow for more effective tailoring of ERAS protocols. Not all patients respond to ERAS interventions. Genetic factors, biomarkers, and clinical characteristics may predict who will benefit most from specific components. As our understanding of these factors grows, we may move toward more personalized enhanced recovery protocols while maintaining the systematic, evidence-based approach that makes ERAS effective [51].

Further research is critically needed. Although hip fracture ERAS is well established, many other trauma types lack robust evidence. Further studies examining enhanced recovery in extremity fractures, pelvic trauma, spinal injuries, and polytrauma are required. These studies should include diverse populations, various healthcare settings, and long-term outcome data. Comparative effectiveness research could help identify which ERAS components are most impactful for different injury types, allowing for more efficient protocol design [52].

Implementation science, the systematic study of methods to promote the integration of research findings into clinical practice, is essential for the sustained success of ERAS. The efficacy of ERAS protocols is well established; however, the remaining challenge lies in achieving consistent and widespread implementation across diverse healthcare settings. Research examining implementation strategies, barrier mitigation, sustainability, and spread can accelerate ERAS adoption. Learning from institutions that have successfully implemented enhanced recovery and understanding why some implementations fail while others succeed will help the field to mature [53].

Global perspectives will enrich the ERAS development. Most ERAS research originates from high-income countries with abundant resources. As trauma incidence is often higher in low- and middle-income countries, developing adapted protocols that work in resource-limited settings is essential for improving patient outcomes. These adaptations may focus on low-cost interventions and creative problem-solving, potentially teaching us valuable lessons that are applicable even in resource-rich environments [54].

Machine learning and artificial intelligence may contribute to ERAS optimization. These technologies can analyze vast datasets to identify optimal protocol components, predict patients at the highest risk for complications, and suggest personalized interventions. While we are still in the early days of artificial intelligence in healthcare, its potential to enhance recovery deserves exploration [55].

Integration with trauma systems represents an exciting opportunity. Currently, most ERAS protocols begin when patients arrive at definitive care facilities. However, enhanced recovery principles could potentially extend backward into pre-hospital care and forward into rehabilitation and long-term recovery. Creating truly comprehensive pathways that span the entire trauma care continuum could further improve the outcomes [56].

## Conclusions

ERAS protocols represent a significant advancement in trauma care, especially in hip fractures. Despite compelling evidence showing improved patient outcomes and healthcare efficiency, implementation remains challenging due to resource constraints, knowledge gaps, organizational barriers, and the inherent unpredictability of trauma care, requiring dedicated MDTs, commitment, and clear protocols. As the evidence base expands to other trauma types, including extremity fractures, and implementation science advances, ERAS has the potential to become the standard of care for trauma patients worldwide. Future directions include technology integration through wearable devices and telehealth, precision medicine approaches to tailor interventions, and adaptation for resource-limited settings.
